# A case report of anaphylaxis induced by topical use of extract of *Cynoglossum wallichii var. golchidiatum*

**DOI:** 10.1016/j.amsu.2022.104206

**Published:** 2022-07-14

**Authors:** Himal Bikram Bhattarai, Sangam Shah, Anup Panthi, Gyabina Maharjan, Ayusha Subedi, Rosy Dhakal, Aakash Koirala

**Affiliations:** a Gandaki Medical College, Pokhara, Nepal; b Tribhuvan University, Institute of Medicine, Maharajgunj, 44600, Nepal; c Kathmandu Medical College and Teaching Hospital, Kathmandu, Nepal; d Manmohan Memorial and Community Hospital, Jhapa, Nepal; e B.P. Koirala Institute of Health Sciences, Dharan, Nepal

**Keywords:** Anaphylaxis, *Cyanoglossum wallichii*, Nepal

## Abstract

**Introduction:**

*Cyanoglossum wallichii* var. *golchidiatum* is a perennial plant that contains pyrrolizidine alkaloids of many types and used as traditional medicine for wounds, burns, and infections.

**Case presentation:**

We report the first diagnosed case of 50 years old male who presented with anaphylaxis after the use of local herbal remedies (***Cyanoglossum wallichii* var. *golchidiatum***) in his cut injury.

**Discussion:**

Without seeing a doctor, individuals in our region of the world administer herbal plants for conditions like burn healing, infections, and wound healing. As a result, there is a delay in the course of treatment, and the herbal therapies have a number of negative side effects. Numerous academic studies have demonstrated the numerous advantages of herbal plants, but despite these advantages, there is a lack of sufficient scientific evidence and knowledge regarding their use and potential drawbacks.

**Conclusion:**

Non-judicial use of ***Cyanoglossum wallichii* var. *golchidiatum*** may lead to potentially life-threatening condition like anaphylaxis

## Highlights

• ***Cyanoglossum wallichii*** is used as traditional medicine for wounds, burns, and infections.
• Topical use of *Cynoglossum wallichii* var. *golchidiatum* plant's extract over a cut injury causes anaphylaxis.

## 1 Background

Anaphylaxis is a potentially life-threatening systemic allergic reaction caused by type 1 hypersensitivity reaction involving one or more organ system that typically occurs within seconds to minutes of exposure to the anaphylactic trigger, most often a drug, food or hymenoptera sting [1]. It can also occur after exposure to certain herbal remedies. *Cynoglossum wallichii* var. *golchidiatum* is a perennial plant of hot and temperate region of Asia including Nepal. The plant contains pyrrolizidine alkaloids of several types and is used as traditional medicine for wounds, burns and infections [2]. Here, we report probably the first case of anaphylaxis after topical use of this plant's extract over a cut injury. This case has been reported as per SCARE 2020 guidelines [3].

## 2 Case description

A 50 years old male vegetable farmer in Nepal developed sudden sensation of flushing and rash all over the body and an episode of vomiting immediately after he squeezed a few leaves of the plant *Cynoglossum wallichii* var golchidiatum over a minor skin slit sustained on a finger during work in his farm. A few seconds later he slumped over and was found to have no pulse by bystanders. Chest compressions were initiated immediately in the field. After about a minute of chest compressions, the patient had partial recovery of consciousness, and he was brought by an ambulance to a hospital nearby. He had no significant prior medical history, nor any history of allergy to medication or other substances. He was not taking any medication.

At presentation to the hospital, he was drowsy and tachypneic with blood pressure, oxygen saturation and heart rate 80/40 mmHg, 96% on room air and 120/minute respectively. There was no wheezing on chest auscultation. He was treated with a reduced dose of epinephrine along with methylprednisolone 1mg/kg, antihistamines, intravenous fluid, and oxygen therapy. He was admitted to the intensive care unit (ICU) for close monitoring. He gradually became fully conscious over a few hours and had no further episode of rash or other discomfort. He was discharged to home the following day.

## 3 Description of herbal product

Kingdom: Plantae.

Phylum: Tracheophyta

Class: Magnoliopsida

Family: Boraginaceae.

Genus name: *Cynoglossum* L.

Species: *Cynoglossum walichii* var*.golchidiatum*.

## 4 Discussion

Natural medicine is generally considered safe with few side effects. However, some of the adverse drug reactions (ADRs) are revealed with extensive use of natural medicine. Most common reactions are allergenic reaction, toxic reaction, and life threatening anaphylaxis [4]. The increasing popularity of herbal plants to enhance the health status is growing in this advanced world. People all over the world should be concerned about this serious issue. Moreover, there is a great role of a botanist to guide for identification and medical benefit of herbal plant and to aware people about the crucial harm caused by such herbal remedies.

The extract of ***Cyanoglossum*** in phytochemical study had shown the presence of alkaloids, coumarins, flavonoids, glycoisdes, phenols, tannins, and xanthoproteins which shows the hepatoprotective, antioxidant, antifertility, antihyperlipidemic, and antidiabetic activity [5,6]. During the toxicological evaluation of *Cyanoglossum*, liver showed mild and moderate fatty degenerative changes, vacuolization of sub mucosal layer of uterus, and decreases spermatogenesis [7]. However, ***Cyanoglossum*** has rarely been reported as a sensitizer for phototoxic reaction, allergic dermatitis, urticaria, asthma exacerbations, and anaphylaxis.

The problem we are facing in our part of world is people administer herbal plants for various purposes like wound healing, infections or burns without consultation of a physician. Therefore, there is delay in treatment and they face various side effects of the herbal remedies. Many literatures have shown multiple benefit of herbal plant but despite such benefit there are no scientific data and adequate information about the use and potential side effects.

Despite such incident people are still using such product without consultation of physician. Allergists and immunologists need to be more knowledgeable about the herbal product and educate people regarding the benefit and side effects of the herbal products**.**

## 5 Conclusion

***Cyanoglossum wallichii*** although having variety of benefits as hepatoprotective, antioxidant, and antidiabetic effects, it has huge potential side effects such as phototoxic reaction, allergic dermatitis, urticaria, asthma exacerbations, and anaphylaxis. Non-judicial use may lead to potentially life-threatening condition. Hence, further studies are needed to explore this issue fully.

## Ethical approval

None.

## Sources of funding

No funding was received for the study.

## Author contribution

SS, AP, and HBB wrote the original manuscript, reviewed, and edited the original manuscript. GB, AS, RD, and AK reviewed and edited the original manuscript.

## Declaration of competing interest

Authors have no conflict of interest to declare.

## Registration of research studies

1. Name of the registry: None
2. Unique Identifying number or registration ID: None
3. Hyperlink to your specific registration (must be publicly accessible and will be checked):

## Guarantor

Dr. Himal Bikram Bhattarai.

## Consent

Written informed consent was obtained from the patient for publication of this case report and accompanying images. A copy of the written consent is available for review by the Editor-in-Chief of this journal on request.

## Provenance and peer review

Not commissioned, externally peer-reviewed.
